# Academic Teachers about Their Productivity and a Sense of Well-Being in the Current COVID-19 Epidemic

**DOI:** 10.3390/ijerph19094970

**Published:** 2022-04-19

**Authors:** Grażyna Bartkowiak, Agnieszka Krugiełka, Sebastian Dama, Paulina Kostrzewa-Demczuk, Elżbieta Gaweł-Luty

**Affiliations:** 1 Faculty of Humanities and Social Sciences, Naval Academy in Gdynia, 81-127 Gdynia, Poland; g.bartkowiak@amw.gdynia.pl (G.B.); s.dama@amw.gdynia.pl (S.D.); e.luty@amw.gdynia.pl (E.G.-L.); 2 Faculty of Engineering Management, Poznan University of Technology, Skłodowska-Curie Square, 60-965 Poznań, Poland; agnieszka.krugielka@put.poznan.pl; 3 Civil Engineering and Architecture Department, Kielce University of Technology, 25-314 Kielce, Poland

**Keywords:** remote teaching, digital competences, academic teachers, COVID-19, quality of working life, mental well-being, teacher work productivity

## Abstract

This article looked at the situation of university teachers in Poland during the COVID-19 epidemic as a result of their need to work remotely. The study was conducted in the first stage (I) on 21 academicians and in the second stage (II) on 18 academicians. The study was conducted to determine the level of productivity of the study group in their online learning competencies in relation to their well-being, as well as during the height, and weakening of the epidemic. The results of the survey conducted, especially during the height of the epidemic, indicated varying levels of self-evaluation of their productivity in relation to their digital competencies linked to the need for them to work remotely, which also affected their psychological well-being. Several cases of respondents indicated a negative assessment of their own productivity, and thus their quality of work life and sense of psychological well-being. However, some respondents, on the contrary, showed a desire to work, the need for self-improvement, and to continue their mission of teaching.

## 1. Introduction

This article considered the question of the functioning of university teachers and their productivity in connection with the need to work remotely and the impact of this situation on their assessment of their professional life and sense of psychological well-being during the COVID-19 epidemic. The basis for consideration of this issue is the qualitative analysis of the results of the study, and the fact that the coronavirus epidemic has changed the functioning of this selected professional group, as well as the entire labor market in Poland [1].

Accordingly, an epidemic emergency was declared on 12 March 2020, and an outbreak was declared on 20 March 2020 [2]; it has also affected the working conditions in Poland, of every professional group, thus worsening the health and well-being of each employee [3,4,5].

Hence, the article addressed two aspects of academic teachers’ working conditions, i.e., their effectiveness and psychological well-being, and thus their subjective assessment of their quality of life in conjunction with the need to work remotely.

For this reason, the conducted study illustrates the changes that are currently taking place in the space of the work environment of university teachers and forces the formulation of a question about the status of universities in the era of global changes that the situation of the COVID-19 epidemic has greatly accelerated. Thus, on the one hand, it raises the question about the readiness of the academic staff to deal with the problems that universities faced, in their time, when the Internet appeared [6]. However, the current situation also raises questions about the functioning of the education system in Poland, especially at the level of higher education, in times of global social, climate, and economic changes, with particular emphasis on the development of information and communication technologies (ICT) [7].

### Teachers in the Situation of the Coronovirus Epidemic

The situation of the COVID-19 epidemic in the world, as well as in Poland, forced changes in the functioning of many professions, including academic teachers. Taking classes online [8] has forced many of them to acquire new digital competencies. Competence of this type implies a construct related to the cognitive sphere that allows the use of the various tools of telecommunications technology to handle information that can be obtained from training in the use of electronic devices and the software used [9]. For this reason, it can be said that a kind of experiment was undertaken [10]. Currently, they were assigned to a specific social group, e.g., IT specialists, but along with their functioning in the information society, they have become one of the elements of social life [11].

Due to this situation, within just a few days Polish universities were forced to take decisive action and implement the so-far unused on such a large scale core curriculum using online learning. The workload of academic teachers increased significantly, especially related to the preparation of classes and their introduction to educational platforms and educational applications, which was contrary to the already proven methods of knowledge transfer and its verification. However, not only academics but also universities, especially in terms of technology and fundings, were not ready to cope with the new conditions, specifically regarding having educational platforms, procedures, and technology related to GDPR compliance. This situation required a multiplication of efforts to overcome the existing problems, including increased effort and excessive workload on the part of academic teachers, multiplied openness to the needs of students, and greater determination to achieve the desired goals.

Consequently, the work life of the study group, relationships with colleagues, supervisors, and others in their work environment changed. Hence, at times, a shift in the professional hierarchy has become noticeable. In contrast, the restrictions put in place due to the epidemic contributed to a metamorphosis in the psychosocial functioning of university teachers. This became particularly difficult for extroverted individuals, where unprocessed traumas at home or work exacerbated intra-group conflicts, thus collapsing the boundaries between work and life [12,13].

The occupational situation of university teachers in relation to their relationship with the environment can be analyzed similarly in terms of popular stress theories [14,15]. However, it must meet the criteria for the sake of digital competence, which may be too difficult in the current situation of the epidemic and the conditions created by it [16]. Hence, every employee should receive support from their supervisors and colleagues at work. However, during the necessary remote work, such an opportunity was significantly limited. Additionally, there was also an imbalance between the effort put into the work and the possibility of obtaining a certain reward for it [17] and, on the other hand, there was an imbalance between the demands at work and the expectations of reward for them [18].

The situation of the studied group was aggravated by the fact of discomfort with health security, which, on the one hand, was intensified by the flow of information coming from the mass media or from people in the closest environment, presenting a situation of threat to health and sometimes even life.

## 2. Theoretical Considerations and Definitional Arrangements

To conduct relevant analyses in the presented study regarding the functioning of university teachers in Poland during the COVID-19 epidemic and the necessity for them to undertake remote work, two categories were created, i.e., teacher productivity and sense of psychological well-being [19]. Accordingly, two concepts were created:

- teacher productivity: a Level of Productivity (LOP);
- mental well-being: Mental Well-Being (MWB).

### 2.1. Teacher Work Productivity (LOP)

The results are presented as a learning outcome, where students or teachers achieve their goals [20].

Conducting a study on teacher performance evaluation is not an easy task. For this purpose, appropriate categories were created. However, when describing teachers’ productivity, some researchers refer to several significant factors such as preparation time, students’ competencies, and teachers’ level of preparation for teaching [21,22]. On the other hand, as individual researchers point out, there is a significant difficulty in defining teacher work. This is because, on the one hand certain institutions set standards and legal rules related to teaching. On the other hand, there is also a certain mission associated with this profession. However, many researchers also focus on subjectivity in defining the above criteria [23]. Therefore, in this publication, productivity will be understood to mean its sense as popularized in OECD countries, where it means: An assessment of the percentage of time or number of days one has (or has not) been productive or functioning well while at work, which could include a specific connection to health (i.e., problems with reduced productivity/functioning due to health problems) [24]. The above definition of productivity is far from its original understanding grounded in economics already known since 1766 and which É. Littré (1883) directly related to the ability to produce [25]. A similar understanding of productivity could be found in the thought of D. Sumantha, D.S. Sink [26], or A. Lawor [27]. Such an understanding of productivity would be too narrow for the phenomenon under study. Consequently, a much more subjective and broader definition was adopted, referring to the environment (as a whole) in which respondents live, and in relation to their health, as pointed out by, among others J. W. Frank [28]. As the COVID-19 epidemic has necessitated the need for academics to work remotely, subcategories of self-reported knowledge regarding the use of information and communication technology by the respondents were used in the study presented here. For this reason, the assessment of self-competence in the use of digital tools was in the next stage of the study as an indicator of the work of the studied social group.

### 2.2. Mental Well-Being (MWB)

When referring to describing the issue of health, it is impossible to refer to its definition. According to the world health organization, health is: a state of complete physical, mental, and social well-being, not just the absence of disease or disability" [29]. The relationship between physical and mental health according to the aforementioned aspects can be characterized as a systemic approach, where each single factor influences the next, both as cause and effect [30]. According to many psychologists, psychological well-being involves the study of the growth of factors such: optimism, courage, work ethic, appearance, anticipation, interpersonal skills, and others more than it relates to the elimination of specific social problems. 

This type of approach was represented by humanistic psychology, initiated by A. Maslow and later developed by M.E. Seligman [31]. This field of psychology focuses primarily on subjective feelings of well-being related to past achievements, as well as hope and optimism related to the future and experience of the present [32]. For this reason, in studies dedicated to the study of well-being, less emphasis is placed on specific disorders or dysfunctions and more on psychological well-being [33].

Referring to the assumptions of positive psychology (including eudaimonism) represented by M.E. Selingman [31,32,33,34], well-being is a balance between work and private time, acting in accordance with one’s self, psychological balance, and self-actualization based on a recognized value system [35,36]. The best-known model of mental well-being is the six-dimensional C.D. Ryff model [37]. It consists of the following elements:

- self-acceptance;
- positive relations with others;
- autonomy;
- environmental mastery;
- purpose in life;
- personal growth.

In the model presented above, it is important to note the existing relationship between sense of psychological well-being and quality of work life. It is difficult to unambiguously determine the relationship between these phenomena, although most often the two coexist. Moreover, according to some psychologists and other researchers, mental well-being refers to a subjective sense of happiness, including job satisfaction, which is a causal dimension of well-being [38]. On the other hand, a factor that increases employee productivity is often job satisfaction as an expression of psychological well-being [39,40].

## 3. Research Method

In the conducted research, the qualitative method of research was used [41]. It included the following research problems, based on the research objectives listed below. The tool used was an in-depth interview based on an interview questionnaire consisting of eight structured questions, also presented below. In order to achieve the stated research objectives, the following research problems were created:

- How do academic teachers assess their competences in the use of their digital competences in the situation of the necessity of online teaching in the phase of epidemic COVID-19 development and in the phase of its silence? (P1);
- How do academic teachers define their level of mental well-being during the epidemic development phase and during the calm phase? (P2).

In order to obtain an answer to these formulated issues, it was necessary to operationalize the measurement partly using the previously mentioned model of C.D. Ryff 

For the purpose of the research project, an in-depth interview was planned, for the needs of which the following instructions were arranged:

1. How do you rate your level of knowledge of the software used for remote work?
2. How do you feel about working remotely?
3. To what extent relationships with friends and other people are a condition for feeling mental well-being?
4. How can you define your attitude to the world?
5. What values do you consider important?
6. What life goals would you like to achieve in the near future?
7. How do you assess your level of mental balance? How do you manage to maintain balance between professional work and family responsibilities and free time?
8. What does personal development mean for you and to what extent do you implement it?

On the other hand, regarding the procedures related to the survey conducted among university teachers, the procedure was as follows. The answers to the above questions were conducted from May to November 2020, where in the first stage (I) 21 academic teachers participated, where all of them were teaching remote classes at that time. This group consisted of 12 women and 9 men. The age of the participants ranged from 31 to 67 years ($\bar{x}\;\;®$ = 52.04). In the second stage of the study (II), 18 university teachers, not including two women and one man, participated. The age of the respondents in this group ranged from 35 to 67 years ($\bar{x}\;\;®$ = 58.56). The sample was purposively selected.

Regarding the first stage of the conducted research, 10 people with a doctoral degree, 9 with a habilitated doctor degree, and 2 with a professorial degree took part in it. The respondents represented various fields of science and scientific disciplines. Among them there were people representing social sciences and humanities (11) and natural sciences (10). As regards the nature of the university, among the respondents there were lecturers from public (14) and non-public (7) universities. On the other hand, in the second phase of the study, which took place in July 2021 after the third wave of the epidemic in Poland, the same people participated in the study, except for two who could not participate for reasons beyond their control.

The first stage of the research involved both persons (10 people) with a doctoral degree, and with a habilitated doctor (9) and the title of professor (2 people). The respondents represented the following scientific disciplines: humanities and social sciences (11) and polytechnic sciences (10). Moreover, the respondents were employees of public (14) and private (7) universities. In the second phase of the study, which took place in July 2021, the same people participated after the third wave of the epidemic, except for two persons who, for random reasons, could not participate in it. Similarly, as in the first stage, also in the second stage of the survey, teachers representing social humanities (10 people), natural sciences (8 people), as well as being employees of public (12) and non-public (6) universities took part. In addition, with regard to their academic degree, 9 PhDs, 8 habilitated doctors, and one professor participated in the study. The study conducted was longitudinal in nature. The existing interval between the first stage of the study and the second was not long. Specific ranges of questions before were coded before the survey was conducted. Thanks to this it was possible to obtain results on the basis of specific categories, which allowed us to operationalize the obtained research material.

### Research Ethics

In the study conducted, the ethical principles applicable to the conduct of such activities were followed. During the survey, the respondents were informed about the purpose of the survey, how the survey might affect them and their life, and how the results would be used. They were informed of the anonymity of the survey and that the security of the data obtained was assured [42]. At any time, any of the subjects could opt out of participating in the study. Moreover, the survey conducted in no way violated the freedom or morality of the respondents [43].

## 4. Research Results

### 4.1. Assessment of Level Own Productivity (LOP) by Academic Teachers

The results of the research presented below contain data on the subjective assessment of the level of teachers’ productivity in terms of knowledge and use of information technology in remote work. The information is presented in Table 1, which presents the subcategories of own assessment (LOP) of academic teacher’s own work productivity during the remote teaching at epidemic of COVID-19 (two stages of research). Source: own research.

The study presented above addresses the first problem, and it concerns the use of digital competences by academic teachers; in the situation of the necessity of remote learning during the epidemic development phase and in the silence phase (P1), the level of academic teacher productivity (LOP) both in the first (I) and in the second study (II) can present three types of characteristics based on successively occurring specific attitudes. The first type of positive evaluation was related to teachers who showed great interest in remote work as well as the willingness to continue developing their digital competences in connection with the necessity to remote work. They were presented a positive attitude towards remote learning of students (Level of Productivity 1) (LOP1).

The second type were people who assessed their digital competences well and wanted to maintain the obtained level of these competences, and even develop them (Level of Prodictivity 2) (LOP2). The third group consisted of teachers who were much less involved in expanding their media competences and treated remote work as an unacceptable necessity. The teachers in this group drew attention to the negative aspects of many hours of work in front of the computer and did not show any willingness to further develop their digital competencies (Level of Prodictivity 3) (LOP3). As shown by the obtained data, the attitude to acquiring competences is correlated with the age of people participating in the research (these are people over 60 years of age). The indicated participants of the research are much less positive about expanding their digital competences and assess their productivity as their colleagues, i.e., younger academic teachers (Level of Prodictivity 3) 

It can be assumed that the attitudes of teachers towards remote work revealed in the research and the observed prices of their own productivity result from the diversified motivation to acquire new media competences in this area.

Comparing the statements from the period of the epidemic development and the period of silence (Level of Prodictivity 3) (LOP3) special attention deserves not so many expressions that can be assessed as a continuation of the opinions inspiring to act presented earlier, but those that indicate the overcoming of the existing aversion and decide to learn and improve media literacy.

### 4.2. The Level of Mental Well-Being in the Group of Surveyed Teachers Based on Their Self-Assessment

In the presented research, the assessment of mental well-being is treated as an element of the subjectively assessed quality of life, about which the respondents expressed their opinions during the in-depth interview. These statements indicated a differentiation of this opinion (which results from the analysis of the adopted assessment categories) depending on demographic characteristics and personality. As has been observed, the formulation of previously established life goals played an equally important and at the same time differentiating role. The Table 2 presents the subcategory of the assessment of the experienced well-being (*MWB*) by academic teachers in connection with taking up remote teaching during the epidemic of COVID-19. 

As shown in the data presented in Table 1, a detailed analysis of the psychological well-being assessed by the teachers participating in the interview showed reduced psychological well-being caused by the inability to maintain the current level of social relations (Mental Well-Being 1) (MWB1), difficulties in maintaining balance between work and private life (Mental Well-Being 3) (MWB3), depressive (Mental Well-Being 5) (MWB5) sense of threat to one’s own health and the life of relatives (Mental Well-Being 4) (MWB4) more common in people aged 60 and over; inability to perform job related to non-personal goals (Mental Well-Being 2) (MWB2) and life frustration difficult to identify, possibly due to personality conditions, and low tolerance to all adversities (Mental Well-Being 3) (MWB3). The respondents’ statements also indicate another category of experiencing well-being: the "specific stability" of this feeling, which occurs regardless of the epidemic (Mental Well-Being 6) (MWB6). This indicates the dynamic activity of a certain group of academic teachers and their commitment to achieving life goals (it can be assumed that also comprises professional goals).

Proposals to deal with low mental well-being as a remedy for the inconvenience of the COVID-19 epidemic are noteworthy. These include life values related to relationships with friends (Mental Well-Being 7) (MWB7) and commitment to numerous duties that will be highly absorbed mentally, which in a way makes you forget about the threat (Mental Well-Being 8)(MWB8) and focus on competence development (Mental Well-Being 9) (MWB9).

Summing up, the assessments of academic teachers can be divided into positive (people in working age) and negative (people in post-working age). It is also related to the problem (P2) which concerns mental well-being (Mental Well-Being) (MWB). Professional activities and the support of relatives made it possible not to collapse as a result of the epidemic. Nevertheless, all respondents indicated that they felt the stress caused by the situation.

In the second stage of the research (II), some people indicated positive aspects of the situation in their statements. These people emphasized the significant frame of well-being as a result of establishing social contacts (Mental Well-Being 1) (MWB1). The second group of respondents included people who showed an improvement in well-being. Although the subjects suggested an improvement in their well-being, they did experience a persistent sense of insecurity (Mental Well-Being 2) (MWB2) and individual inconveniences resulting from the hybrid learning system (Mental Well-Being 3) (MWB3). The third group of people was characterized by an insignificant improvement in well-being along with a persistent sense of insecurity and threat (Mental Well-Being 4) (MWB4) or a sense of instability and a growing sense of threat, which resulted in a general reduction in the sense of well-being (Mental Well-Being 5) (MWB5).

Another group of respondents comprised people who, as a result of the situation, devoted themselves to specific duties (Mental Well-Being 8) (MWB8) or interests (Mental Well-Being 9) (MWB9), which allowed them to maintain a satisfactory state of well-being. Moreover, these respondents (Mental Well-Being 9) (MWB9) emphasized the benefits of the epidemic mainly in the form of recognition and development of professional interests. Compared with the period before the epidemic, as later, a certain type of activity allowed them to refresh or awaken previously unrecognized interests and passions. As can be expected, these interests will positively shape their sense of well-being.

## 5. Discussion

Research shows differences in the academic teachers’ assessment of their productivity resulting from digital competences for remote work. There are also visible differences in the assessment of the quality of professional life and the sense of mental well-being resulting from the COVID-19 epidemic. The survey showed both negative and positive opinions about one’s own productivity, quality of working life, and the sense of mental well-being. However, all respondents confirmed the stress related to the COVID-19 epidemic. Some academic teachers showed pro-social values: the mission of being a teacher helped them overcome various inconveniences related to the situation.

Some of the respondents (aged over 60) showed concern for the effectiveness and quality of youth education, regardless of the inconvenience and difficulties associated with changing the teaching conditions. Some also emphasized the support of close people, friendly relations, and a sense of closeness with other people as a remedy for coping with stress and threats to health and life.

Some academics have shown a desire to improve their digital competences and professional development because of the epidemic situation. Other respondents tried to deal with stress by engaging in numerous responsibilities, which turned out to be a positive strategy.

The analysis of the research results proved a lower level of self-assessment of the quality of working life, sense of well-being and productivity in people aged 60 and over compared with younger colleagues, although it is positively surprising that in the second stage of the study (II) there were people over 60 years of age who expressed a desire to improve their digital competencies. Regardless of whether it is only a declaration, in a situation where these people assumed that there would be no need to work remotely, and that teaching students would be carried out in a stationary manner, the observed statements are optimistic. Moreover, it should be remembered that there is a high probability that these statements may not represent the attitude towards broadening their competencies of academic teachers aged 60 plus as a professional group.

The results of the research carried out in comparison with the results of previous research by other scientists are partially confirmed, especially in relation to the first stage of the research (A). However, in the second stage of the study (II), the obtained results do not fully coincide with their previous counterparts.

As a result, in the research presented in the work of Parker with the team [44], academic teachers were not very well assessed by students in relation to their digital competences. In the study of American researchers, students emphasized that academics are less involved in preparing online classes than they are in the case of offline classes [45]. Regardless of this fact, the authors of the article adopted the understanding of teacher productivity as a completely subjective category, and the results of the research do not coincide with the self-esteem of the respondents in the qualitative research conducted for the purpose of this article. On the other hand, it is even more optimistic that the respondents 60 years and older declared their willingness to improve their digital competencies. Moreover, in connection with the subcategory of self-assessment (LOP), the above declarations of the respondents showed, represented in the first stage of the study (I), similarly to Huk’s research [46], that due to the changes taking place and the inability to adapt to them, people in the above-mentioned age show less activity in particular areas of social life. In the second stage of the study (II), this result did not fully cover the presented theory of withdrawal. The explanation of presented statements does not confirm the data obtained in qualitative research by Machielse and Duyndam [46], which, according to the theory of Giddens’ structuring [47,48], showed that the elderly chooses behavioral strategies that imply the preservation of resources and a sense of security. At the same time, they are consistent with the research carried out by Wanka [49], indicating that advanced age and social stereotypes that are not very positive, and which result from it, can be resisted if they show specific activity themselves, leading to an increase in their own well-being. Moreover, the AGIL (adaptation (A), goal attainment (G), integration (I), latent pattern maintence (L) analytical scheme and the structural theory of T. Parsons explain the obtained test result about the activity of respondents 60 years old and older [50], especially in their level of productivity in learning to use the new software (LOP2). As part of adaptation, achieving goals, a sense of internal integration, as well as latency within social groups in which the respondents participate, the theory of action based on the theory of evolution presented by the American sociologist seems to emerge, where the existing differentiation between the systems represented by the respondents and their environment, a specific functional dependence is also created in relation to the created patterns in connection with the situation of remote work. New principles and mechanisms of integration are also emerging, which clearly differ from the existing ones and are deepening at the present stage [51] of the research. Thus, the ability to survive in the existing diversified system is created. On the other hand, further research and observations would allow for the validity of the presented thesis.

In addition, it was observed that demonstrating the motivation to take up activity in social life coincides with Huk’s theory of activities [52]. Therefore, it can be expected that people above working age and unable to fulfill themselves in specific areas of life, also in education through distance teaching, try to fulfill their own interests in art and cooking. This was confirmed by the results of the first (I) and second stage (II) of the study.

In addition, research during the COVID-19 epidemic has been found to show that online mentoring training is effective in increasing the motivation and online teaching of science teachers’ skills during the COVID-19 period [53]. Hence, it can be expected that technical support in remote learning for selected surveyed academic teachers, regardless of the period of the epidemic, would bring positive results. Similar conclusions were reached by other researchers who, apart from the need to acquire technical skills in distance teching by academic teachers, emphasized the importance of supporting competences in increasing wellbeing [54].

This conclusion also fully applies to the research analyzed in this article.

The conducted research showed different reactions to the stress of perceiving it and ways of coping with the situation of the COVID-19 epidemic related to the threat to health and requiring the acquisition of new competences of remote teaching. The reaction to the described stress also turned out to be varied depending on the age and experience of academic teachers. It could be expected that in old age people have more sophisticated strategies for coping with stress [55,56,57,58,59]; however, the obtained data, as was the case in the studies of such authors as Goel Verma [60], confirmed a more intense and emotionally burdensome perception and assessment of the stressful situation in the older age group. In addition, as depicted by the survey results, younger respondents showed less trepidation about the COVID-19 situation put them in. They were excited about the situation of being forced to work online because they liked it, and they also wanted the change that, in terms of education (Level of Productivity 1, type I, example 1) (L_op1I1_), distance learning had already made in other countries around the world (Level of Productivity 1, type II, example 1) (L_op1II1_). On the other hand, because they were observed with their daily life, they did not have any reflections related to the situation related to COVID-19 (Mental Well-Being 8, type I, example 1)(M_WB8I1_), (Mental Well-Being 8, type II, example 1) (M_WB8II1_-Level of Well-Being 8, type II, example 1—represent respondent who maintains a balanced state of well-being by indulging in daily chores). In contrast, those above working age and mobile age did not share such excitement about the need to work remotely (Level of Productivity 3, type II, example 3) (L_op3II3_), (Level of Productivity 3, type I, example 2) (L_op3II2_), or were accustomed to or convinced of the greater effectiveness of teaching through unmediated contact with students (Level of Productivity 2, type II, example 1) (L_op2II1_) (Mental Well-Being 1, type I, example 1) (M_WB1I1_). However, there were also people of the age presented above who were eager to continue teaching online in hybrid learning (Level of Productivity 2, type II, example 1) (L_op2II1_), (Level of Productivity 2, type I, example 1) (L_op2I1_) or who were either distraught about the situation that the COVID-19 epidemic had put them in (Level of Productivity 3, type I, example 1) (L_op3I1_), (Level of Productivity 3, type I, example 2) (L_op3I2_), (Mental Well-Being 2, type II, example 1) (M_WB2II1_), (Mental Well-Being 3, type I, example 1) (M_WB3I1_), (Mental Well-Being 3, type II, example 1) (M_WB2II1_), (Mental Well-Being 4, type I, example 1) (M_WB4I1_), (Mental Well-Being 5, type I, example 1) (M_WB5I1_), (Mental Well-Being 5, type I, example 2) (M_wb5I2owII5_), or that it was changing their lives (Mental Well-Being 9, type I, example 1) (M_WB9I1_), (Mental Well-Being 9, type II, example 2) (M_WB9II2_), (Mental Well-Being 9, type 1, example 2) (M_WB9I2_), (Mental Well-Being 9, type II, example 1) (M_WB9II1_). The attitude that differed between the older and the younger respondents who were of prime working age had no reflections related to the COVID-19 situation, did not manifest health problems, and did not show any fear, anxiety, or changes that this situation could bring about in their lives. Therefore, they were the ones who exhibited higher efficacy and high mental well-being compared with older respondents. In the study of Joja, Lazaro, Garcia-Zapirian, Gonzales, and Urizar, negative conflicts between students and lecturers were manifested by students, equally in the aspect of efficiency and well-being [61], but it is more common for younger workers to be more willing to work online or in a hybrid system than older workers. Referring to the issue of teachers’ well-being in the pre-pandemic and post-pandemic time according to the study Alves, Lopes, and Precisio, teachers were shown to have higher well-being and effectiveness before the COVID-19 epidemic than during the outbreak [62]. 

However, in addition to mental health, as one part of the study showed, the severity of the necessary remote work of the university teachers surveyed could also be observed in the somatic dimension. In 1999, K.S. Young indicated that prolonged use of the Internet may adversely affect human health. According to her, this type of activity can cause sleep disruption, cause severe fatigue, weaken the immune system, form negative eating habits, lead to body hygiene disorders, cause severe back pain, stimulate carpal tunnel syndrome, and stimulate headaches and eye fatigue [63,64]. In the conducted interviews the respondents pointed out to several of the mentioned symptoms, which were caused by prolonged staying in front of the computer and using the Internet. Respondents complained of damaging their eyesight by being in front of the computer for long periods of time back pain (Mental Well-Being 4, type I, example 1) (M_WB4I1_), feeling tired (Level of Productivity 3, type II, example 3) (L_op3II3_), feeling neurotic (Mental Well-Being 5, type I, example 1) (M_WB5II1_), or feeling generally impaired (Level of Productivity 3, type I, example 3) (L_op3I3_). The COVID-19 epidemic condition also became a factor that ushered in a state of "new normalcy" [65]. For this reason, it can also significantly affect changes in health in the public sense [66]. However, as also indicated by other researchers, prolonged lockdown had a negative impact on the well-being of the university teachers surveyed, mainly causing a sense of increased stress, and worsening the quality of life. On the other hand, a study conducted on students by Iurcov, Pop and Iorga due to the need for social distance and a non-contact dimension of the class affected their feelings of difficulty with sleep, eating, depression, anxiety, and boredom [67]. On the other hand, it should be emphasized that the above results are significantly different from the results in other sectors of the economy and work that do not require direct contact with the person to whom the services are provided. However, also it should be emphasized that the above results are significantly different from the results in other sectors of the economy and jobs that do not require direct contact with the person being served, such as call centers or other jobs that are performed in an office and do not require intellectual engagement and emotion [68].

These situations in the long run will not only leave a mark on the health of the people who experienced the COVID-19 epidemic, but will also force new standards for preventive health care as well as for the treatment itself, not only as a result of the need to take exceptional precautions, but also as a result of the changes that have taken place in this regard in the economic space, which is linked to health care in both its public and private dimensions. As already mentioned in the qualitative study, in the first stage of the study (I), older people compared with the younger ones were characterized by a lower evaluation of mental well-being. Comparing the conducted research with the research of Prasad and colleagues [69], the results obtained by these researchers did not confirm that stress related to the risk caused by the work situation in remote situations reduces the activity of the surveyed employees and thus their sense of mental well-being. Therefore, based on the conducted research, changes can be noticed in the attitudes of academic teachers in the comparison of the first (A) and second (B) stage of the study. However, the study conducted for this article showed that among Polish academic teachers, as a result of the need to work remotely forced by the epidemic situation, the well-being of most of them, including those over 50, as well as productivity has significantly decreased [70]. Thus, this also translated into the quality of work performed. Besides, as is worth emphasizing, the necessity of increased work in front of a computer, the use of the net and the dominance of the sitting position, especially in the case of people who were already in the pre-retirement age also showed health problems in contrast to people who were under 40. Moreover, the examples presented in the study and the conclusions drawn from it indicated that both older respondents at present and younger ones in a few years will show, especially in the case of the necessity to work online, often in a sedentary position may manifest disease phenomena, which so far have been only a form of pathology, and which over time may assume the dimension of a disease of civilization. For this reason, the presented study contributes a certain value to future research, which concerns health in the individual, but also the public dimension, especially in case of appearance of some new threats related to a new form of epidemic or work in front of a computer.

## 6. Conclusions

As it was mentioned in the first and in the second stage of the study, it is necessary to continue research in the area of the analyzed issues and to compare the research results analyzed in this article with the results of other studies. Therefore, it is necessary to conduct further observations of academic teachers with regard to remote work in the COVID-19 epidemic. It is also worth emphasizing at this point that using a qualitative method in the form of in-depth interview, apart from the cognitive assumptions, also allowed the respondents to gain greater awareness not only around their own experiences in the epidemic era, but also in terms of their own relations with the environment in which they live.

The presented research has some limitations: it covered a small research sample which, despite the qualitative nature of the research, does not allow for the generalization of the results.

The research carried out and the results resulting from them bring the functioning of Polish academics and universities during the coronavirus epidemic closer and will make a certain contribution to the study of this phenomenon and related problems, including in health in private and public dimensions, which will also become the subject of analysis by researchers at home and abroad.

## Figures and Tables

**Table 1:** Subcategories own assessment (LOP) of academic teacher’s own work productivity during the remote work during the epidemic of COVID-19 (two stages of research). Source: own research.

Research Stage	Category of Self-Productivity Assessment in the Necessity of Remote Work	The Symbol of Category of Self-Productivity. Evaluation of Remote Work	Examples of Evaluation of Self-Productivity in Situation of Neccesity for Remote Work in a Predefined Category
I.	This category consists of people who are particularly involved in remote work, and highly value their digital competences as an indicator of their productivity. These people have not yet reached the age of 35, they are trying to further improve their media competences.	LOP1	L_op1I1_: “I’m glad that I can finally do what I like, I’m inventing ways to work in groups on MS Teams. Maybe I am immodest, but I know a few more programs than my colleagues, because I live a bit of it …” (PhD, man, 33).
II.	These individuals continued to demonstrate the determination and willingness to intensively develop their digital competences, and they showed ambition and concern to further deepen their knowledge and skills.	LOP1	L_op1II1_: “I’m going to keep up with our South Korean colleagues. They know 15–20 years more than we do … about the possibilities of remote work. I am interested in it, although I know that this is another challenge…” (PhD, man, 33).
I.	The category of people who assess their digital competences well and at the same time constantly want to improve them and learn new applications useful in remote work. This group of people was aged 36–60.	LOP2	L_op2I1_: ”"Currently I am doing better and worse … I did not know that I would be able to learn so many new things and I am still learning … I try to explain every difficulty and error … and I do it on every occasion…’ (PhD, woman, 55).
II.	People who still wanted to keep or even develop their digital competences.	LOP2	L_op2II1_: “Now this knowledge will be useful and you cannot depart from this form of contact with students … Certainly, new applications will appear in a moment, you need to be up to date …“ (Phd, female, 55).
I.	People who are forced to work remotely but are not interested in learning new types of software and applications, are not at all interested further work remotely. Usually, this group consists of people over 60 years of age.	LOP3	L_op3II3_: “I’m tired of working remotely, I’m just waiting to mix myself up with this activity, it’s not for me.” I know I’m bad at these things, but I have my age too…” (PhD, man, 67). L_op3I2_: “I don’t want anything to do with remote work anymore and as soon as I can, I will ask for stationary classes” (PhD, woman, 60). L_op3I3_: ”Today I am fed up with hours spent sitting in front of the computer. It seems to me that I am constantly doing the same and ruining my health. How many hours can you sit and classes are only in the form of a lecture, I can’t do anything else” (PhD, woman, 62). L_op3II1_: ”I want to be better prepared for remote work, both for myself and in case there is a need to return to work remotely” (PhD, woman, 60).
II.	People who paid attention to the negative aspects of many hours of work in front of the computer and presented rather a lack of willingness to develop their digital competences. At the same time, there were statements that indicate overcoming reluctance to learn media competences	LOP3	L_op3II2_: ” Finally, normality will return, I am fed up with destroying my eyesight for several hours a day on the screen … I have learned something, but I would prefer not to use these forms of teaching students” (PhD, man, 67). L_op3II3_: “Now I look at remote classes a little differently and if they will be in hybrid mode, I will try to prepare classes in groups and forums. I can’t do it yet, but I will learn it" (PhD, woman, 62).

LOP1-Level of Productivity 1—represents respondents who accept and are prepared to adopt the situation of COVID-19 epidemic and nessesity of remote work. Lop1I1-Level of Productivity 1, type I, example nr 1 represent respondents who valuate their digital competencies. Lop1II1-Level of Productivity 2, type II, example nr 1 represent respondent who develop digital competencies already possessed. LOP2-Level of Productivity 2—represents respondents who accept and adopt to the situation of COVID-19 epidemic and the nessessity of remote work by improving their digital competencies. Lop2I1-Level of Productivity 2—type 1, example nr 2 represents respondents who accept their digital competence and want to raise their level higher. Lop2II1-Level of Productivity 2—type II, example nr 1 represents respondents who keep their digital competence and want to raise them on higher level. LOP3-Level of Productivity 3—represent respondents who are not accepting the necessity of remote work forced by the situation of COVID-19 and manifest negative feelings about it. Lop3II3-Level of Productivity 3—type II, example 3, represent respondent who represents an incerace of productivity and do not want to work remotely. Lop3II3-Level of Productivity 3—type I, example nr 1 represent respondent who does not want to upgrade their digital competencies related to COVID-19. Lop3I2-Level of Productivity 3—type I, example nr 2 represent respondent who does not want to upgrade their digital competencies and represent a decreased level of productivity. Lop3I3-Level of Productivity 3—type I, example nr 3 represent respondent who does not want to upgrade their digital competencies related to COVID-19 and complains of declining health due to remote teaching and decreased level productivity. Lop3II1-Level of Productivity 3—type II, example nr 1 represent respondent who does want to upgrade their digital competencies related to COVID-19 and represent the decreased level of productivity. Lop3II2-Level of Productivity 3—type II, example nr 2 represent respondent who does want to upgrade their digital competencies related to COVID-19, complains of declining health due to remote teaching, but looks to the future optimistically with the idea of returning to what was before the epidemic situation. Lop3II3-Level of Productivity 3, type II, example 3—represents respondents who are open to learn the teaching during the online learning and represent an increased level of productivity.

**Table 2:** Subcategory of the assessment of the experienced well-being by academic teachers in connection with taking up remote work during the epidemic of COVID-19. Source: own research.

Research Stage	Assessment Category of Experienced Mental Well-Being	Category Symbol	Examples of Statements by Teachers Participating in the In-Depth Interview
**I.**	Decreased level of well-being. Persons assessed as extroverted based on the interview complained about the limited possibilities of social and work-related contacts (a).	MWB1	**M**_WB1**I1**_: “I feel very bad when I can’t meet my friends and students … I can’t imagine it would last longer” (PhD, woman, 56).
**II.**	Improving well-being through socializing (a1).	MWB1	**M**_WB2**II1**_: “It looks like it will finally be normal at last. I am already making up my social arrears, but my colleagues from work have got used to the new reality and do not always want to meet in real life. It bothers me…, but I think it will change” (PhD, woman, 56).
**I.**	People with a reduced level of well-being. A significant par of the interview participants regretted that they could not complete the previously planned research. These people focused on professional values (b).	MWB2	**M**_WB2**I1**_: “It was so important for me to complete the research … I think about the insertion material, which will be sick for several years…” (PhD, man, 62).
**II.**	Some improvement in well-being, persistent lack of safety (b1).	MWB2	**M**_WB2**II1**_: “I think I will be able to finish my project, although my team crumbled a little. I don’t know how much time we have again…until another virus. It is not a comfortable situation” (PhD, man, 62).
**I.**	This category referred to people presenting a reduced level of well-being. Most often it was composed of experienced academic teachers who paid attention to the fact that they were not able to maintain a balance between their personal life and professional work (c).	MWB3	**M**_WB3**I1**_: “I sit in front of the screen all day and sit … and take roots, I have no time for anything … as long as I can do it” (PhD, woman, 60).
**II.**	Some improvement in well-being, further individual inconveniences resulting from the announced hybrid education system (c1).	MWB3	**M**_WB3**II1**_: “I am glad that we have a better situation, but I do not know in what direction the changes will take place. Will it not be the case that I will be “chasing” all day from classroom activities by going to another building, 500 meters away, to my room. It will take more time and nerves” (PhD, woman, 60).
**I.**	Category of people with a reduced level of well-being. Another group of people showed a particularly intense interest and concern for their own health, the health of their children, grandchildren and close friends. Another group of people showed a particularly intense interest and concern for their own health, the health of their children, grandchildren and close friends (d).	MWB4	**M**_WB4**I1**_: “Every day my spine hurts …, my children are in constant contact with clients …, my grandchildren are so sensitive, they can easily get sick …, I don’t see the light in the tunnel…” (PhD, woman, 61).
**II.**	Slight improvement in well-being, persistent feelings of insecurity and lack of safety (d1).	MWB4	**M**_WB4**II1**_: “COVID time is like a war whose effects last for years. We do not know the health effects of suffering from the disease or the consequences of vaccinations. A minimum of 2 years is required for this … The grandson is allergic, the doctors do not know how he will react to the vaccination…” (PhD, woman, 61).
**I.**	The category of people with reduced mental well-being, combined with a negative assessment of their own mental state bordering on experiencing depression. At the same time, these people try to cope with their own difficulties (f).	MWB5	**M**_WB5**I1**_: “I often feel very depressed, and it is really difficult for me … I work in our association … something is always happening … My mental wellbeing is better, but not entirely. I am not sure that the situation is stable…” (Doctor, woman, 67). M_WB5I2_: “Now I understand what the term fragility of life means… I am not sure that the situation is stable …” (Professor, man, 68).
**II.**	Slight improvement in well-being, persistent feelings of insecurity and lack of safety. (f_1_).	MWB5	**M**_WB5**II1**_: ”They inform us that a malignant strain of the virus is developing, which is not affected by vaccination. This could be the end of humanity…” (Professor, man, 68).
**I.**	A group of teachers, with a long experience, reflecting on the meaning of their professional life, who did not notice any changes in their professional life, making a comparison with the period before the pandemic (g).	MWB6	**M**_WB6**I1**_: “I do not see any drastic changes in my life in the COVID period, I do what I always did, I probably like it … and I will continue to do so…” (PhD, man, 67).
**II.**	The same level of well-being	MWB6	**M**_WB**II1**_: “I always thought that whether there is COVID or not, everyone should do their own thing … I do what I did before the COVID and I think it will continue to do so. I am not tormenting myself with what was or what will be” (PhD, man, 67).
**I.**	A group of teachers actively responding to the reality caused by the pandemic. Usually, interviewees pointed to their own ability to overcome difficulties and emphasized the particularly important importance of such values as love, care for loved ones, friendship (h).	MWB7	**M**_WB7**I1**_: “In the current situation, my wife and my closest friends allow me to survive and pursue other goals that are subordinated to permanent values” (Professor, man, 64).
**II.**	Raising the rank of specific values as a factor that gives meaning to life (h_1_)	MWB7	**M**_WB7**II1**_: ”COVID allowed me to reaffirm my values. I believe that love, friendship, a supportive family are the most important things that make sense in life. I think it was an important experience…” (Professor, man, 64).
**I.**	The category of teachers who overcome the negative emotions associated with COVID-19 by sinking into numerous responsibilities. Usually, this group consists of junior academic teachers. These teachers, due to the fact that they are very preoccupied not only with professional duties but also with family duties (this group includes both women and men), give the impression of people less afraid of being infected with the COVID-19 virus that their colleagues are more advanced in terms of professional experience and age. Their lifestyle seems to be more action-oriented than self-reflection (i).	MWB8	**M**_WB8**I1**_: “I don’t have time to think about COVID, my son has an eight-year exam, my daughter is in kindergarten and still on the run… I don’t feel particularly threatened … I really don’t have time to think about why I’m doing this and what’s the point of it” (PhD, woman, 35).
**II.**	Deepening commitment to professional duties (i_1_).	MWB8	**M**_WB8**II1**_: “I don’t know what to put my hands into … I don’t think about a epdemic… I have so many things and responsibilities … and I have to finish the project” (PhD, man 39).
**I.**	A category of teachers who focus on expanding and supplementing their competences. For many of them, learning new things is a factor improving their mental well-being. They are a group of people focused on caring for their personal development not only boiling down to learning new digital competences, but sometimes developing other interests and passions (j).	MWB9	**M**_WB9**I1**_: “Now I cook my favorite dishes twice a week, bake a cake …for which I did not have time before (PhD, man, 41)”. M_WB9I2_: “I bought equipment, a dinghy, I bought a fishing license and I run away from the threat to the lake, sometimes I take my son and a friend … (PhD, woman, 44)”.
**II.**	A group of teachers focus on the development of non-professional interests The realization of non-professional interests is perceived as a return to consciously chosen passions, suspended from the necessity to perform professional work, allowing for self-realization (j_1_).	MWB9	**M**_WB9**II1**_: ”My approach to life so far was only professional work, now I have culinary art and portrait painting. It is a COVID added value. I am happy about that” (PhD, man, 41). M_WB9II2_: “Owe COVID that I returned to fishing, which I did in my youth. This is my passion now. For this reason, we bought a plot of land by the lake. I am afraid that I will pull myself in too much …” (PhD, man, 44).

MWB1-Level of Mental Well-Being 1—represents respondents who are complaining about the limitation of social contacts and work environmental f space. MWB1I1-Level of Mental Well-Being 1, type I, example 1, represents respondents with a low level of well-being and representing the negative emotions. MWB2II1-Level of Mental Well-Being 1, type II, example 1, represent respondent with low level of well-being, but with sense of future change in of situation related to the COVID-19. MWB2-Level of Mental Well-Being 2—represents respondents who have a reduced level of well-being and presenting some improvement of well-being and persistent lack of safety. MWB2I1-Level of Mental Well-Being 2, type I, example 1—represents respondents who are focusing on work and represent the low level of well-being in relation the effect of their work whit the influence the COVID-19 situation on that. MWB2II1-Level of Mental Well-Being 2, type II, example 2—represent respondents with a low level of well-being and worrying about the effect of their work on which have the situation of COVID-19. MWB3-Level of Mental Well-Being 3—represent respondents who present reduced level of well-being in relation to the situation related to the COVID-19 or represent some improvement of well-being announced to the hybrid education. MWB3I1-Level of Mental Well-Being 3, type I, example 1, represent respondent who represents the na-negative emotions and low level of well-being in relation to the situation of COVID-19. MWB3II1-Level of Mental Well-Being 3, type II, example 2, represent respondent worrying about the situation in their work activity in relation to the situation of COVID-19 and with low level of well-being. MWB4-Level of Mental Well-Being 4—represent respondents who present reduced level of well-being due to a threat to one’s health and slight of improvement of well-being with feeling of lack of safety. MWB4I1-Level of Mental Well-Being 4, type I, example 1—represent respondent who are experience harm to their health due to the COVID-19 situation and thus show low levels of well-being. MWB4II1-Level of Well-Being 4, type 2, example 2—represent respondent complaining on their own health and that of their loved ones and showing low well-being in relation the situation of COVID-19. MWB5-Level of Well-Being 5—represent respondents with reduced mental well-being combined with a negative assessment of their mental health and people with a slight improvement of well-being but with feeling of lack of safety. MWB5I1-Level of Well-Being 5, type I, example 1—represent respondent complaining about their mental health in relation of the life situation due to COVID-19. MWB5I2-Level of Well-Being 5, type I, example 2—represent respondent representing the low level of well-being and negative situation due to COVID-19. MWB5II1-Level of Well-Being 5, type II, example 1—represent respondent representing the negative perception of the COVID-19 situation and the low level of well-being. MWB6-Level of Well-Being 6—represent respondents who did not notice any changes in their professional life to the compassion before pandemic. MWB6I1-Level of Well-Being 6, type I, example 1—represent respondent who do not perceive a significant change in their well-being and living situation related to COVID-19. MWBII1-Level of Well-Being 6, type II, example 1—represent respondent who is focused on his work and does not dwell on a particular COVID-19 situation and thus maintains a balanced state of well-being. MWB7-Level of Well-Being 7—represents respondents who are positively reacting of the situation of COVID-19 in relation to the important values related to their relatives and the values which gives to them a meing to life. MWB7I1-Level of Well-Being 7, type I, example 1—represent respondent who maintains a balanced state of well-being in relation to the COVID-19 situation because of his unique relationship with his loved ones. MWB7II1-Level of Well-Being 7, type II, example 1—represent respondent who maintains an equivalent state of well-being due to indulging in positive emotions related to other close people around him. MWB8-Level of Well-Being 8—represents respondents who overcome the negative emotions sinking in numerous responsibilities and avoiding the reflections related with the situation of COVID-19 and in this same way improving their well-being. MWB8I1-Level of Well-Being 8, type I, example 1—represent respondent who maintains a balanced state of well-being with devotion to daily life and activities related to their loved ones. MWB8II1-Level of Well-Being 8, type II, example 1—represent respondent who maintains a balanced state of well-being by indulging in daily chores. MWB9-Level of Well-Being 9—represent respondents who are focusing on expanding their digital competencies or passions and in this same way improving their well-being. MWB9I1-Level of Well-Being 9, type I, exmaple 1—represents the respondent who maintains a balanced state of well-being by indulging his/her passions related to everyday life. MWB9I2-Level of Well-Being 9, type I, example 2—represent the respondent who maintains a balanced state of well-being by indulging his/her passions not related to everyday life. MWB9II1-Level of Well-Being 9, type II, exmaple 1—represents the respondent who maintains a balanced level of well-being by indulging in passions related and unrelated to daily life. MWB9II2-Level of Well-Being 9, type II, example 2—represents the respondent who maintains a balanced level of well-being by indulging in passions related and unrelated to daily life and thereby changing one’s lifestyle.

## Data Availability

The data presented in this study are available on request from the corresponding author. The data are not publicly available due to restrictions privacy.
